# Targeted metabolomics profiling in pregnancy associated with vitamin D deficiency

**DOI:** 10.1186/s12884-024-06454-7

**Published:** 2024-04-20

**Authors:** Xiaogang Li, Zhuoling An, Aimin Yao, Rui Li, Suhan Zhang, Songlin Yu, Liangkun Ma, Yanping Liu

**Affiliations:** 1 Department of Clinical Nutrition, State Key Laboratory of Complex Severe and Rare Diseases, Peking Union Medical College Hospital, Chinese Academy of Medical Science and Peking Union Medical College, 1 Shuaifuyuan Road, Beijing, 100730 China; 2 Biobank Facility, State Key Laboratory of Complex Severe and Rare Diseases, Peking Union Medical College Hospital, Chinese Academy of Medical Science and Peking Union Medical College, Beijing, China; 3 Pharmacy Department of Beijing Chao Yang Hospital, Capital Medical University, Beijing, 100020 People’s Republic of China; 4 Department of Gynaecology and Obstetrics, Shunyi Women’s and Children’s Hospital, Beijing, People’s Republic of China; 5 Department of Obstetrics and Gynecology, State Key Laboratory of Complex Severe and Rare Diseases, Academic Medical Science and Peking, Peking Union Medical College Hospital, Union Medical College, Beijing, 100730 People’s Republic of China; 6 Department of Clinical Laboratory, State Key Laboratory of Complex Severe and Rare Diseases, Academic Medical Science and Peking, Peking Union Medical College Hospital, Union Medical College, Beijing, 100730 People’s Republic of China

**Keywords:** Pregnancy, Targeted metabolomics, Vitamin D, Mass spectrometry

## Abstract

**Background:**

Vitamin D deficiency is common in pregnancy, however, its effects has not been fully elucidated. Here, we conducted targeted metabolomics profiling to study the relationship.

**Methods:**

This study enrolled 111 pregnant women, including sufficient group (*n* = 9), inadequate group (*n* = 49) and deficient group (*n* = 53). Ultra-high performance liquid chromatography-tandem mass spectrometry (UHPLC-MS/MS)-based targeted metabonomics were used to characterize metabolite profiles associated with vitamin D deficiency in pregnancy.

**Results:**

Many metabolites decreased in the inadequate and deficient group, including lipids, amino acids and others. The lipid species included fatty acyls (FA 14:3, FA 26:0; O), glycerolipids (MG 18:2), glycerophospholipids (LPG 20:5, PE-Cer 40:1; O2, PG 29:0), sterol lipids (CE 20:5, ST 28:0; O4, ST 28:1; O4). Decreased amino acids included aromatic amino acids (tryptophan, phenylalanine, tyrosine) and branched-chain amino acids (valine, isoleucine, leucine), proline, methionine, arginine, lysine, alanine, L-kynurenine,5-hydroxy-L-tryptophan, allysine.

**Conclusions:**

This targeted metabolomics profiling indicated that vitamin D supplementation can significantly affect lipids and amino acids metabolism in pregnancy.

## Introduction

Vitamin D is a fat-soluble vitamin, which mainly exists in the human body in two forms, vitamin D2 and vitamin D3. Vitamin D3 accounts for 90 − 95% of the total vitamin D. Vitamin D is converted into 25-hydroxyvitamin D in the liver and kidneys, and then participate in biological processes in vivo. The role of vitamin D is mainly reflected in the following aspects: promote the absorption of calcium and phosphorus in the intestines, maintain bone health; enhance muscle strength and improve muscle endurance; promote immune function and reduce the risk of infection; reduce the risk of diseases such as diabetes, cardiovascular disease, and certain cancers [1, 2]. Vitamin D is speculated to play a role in female reproductive function, since many reproductive organs such as uterus, ovary and placenta express vitamin D receptors. Besides, vitamin D is an immunomodulator, which help to promote immune tolerance pregnancy. Therefore, vitamin D may have an effect on reproductive function and maternal outcomes. Various studies have evaluated the relationship between vitamin D and human fertility. To evaluate the role of vitamin D intake and serum levels on conception of clinical pregnancy and live birth. The researchers prospectively assessed their vitamin D intake and supplements through surveys conducted at baseline, at 3 months, and at 6 months. The results showed that couples who continued the use of recommended daily intake had a higher chance of getting pregnant. Those women with adequate levels of 25(OH)D have a higher pregnancy rate [3]. Multiple studies have shown that infertile women with higher levels of 25(OH)D in plasma and/or follicular fluid are more likely to become pregnant after reproductive assistance procedures such as intracytoplasmic sperm injection (ICSI) or in vitro fertilization (IVF) [4, 5]. Other studies have no conclusive results. As for egg donation therapy, donor women with 25(OH)D levels below 20ng/mL have a lower pregnancy rate than donor women with 25(OH)D levels above 30ng/mL [5]. Vitamin D deficiency is common in pregnancy, however, its effects has not been fully elucidated. Some animal experiments show that impaired fertility and decreased ovarian function are associated with vitamin D deficiency [6]. But, the studies investigating vitamin D deficiency in pregnant women are still lacking.

Metabolomics is a method to quantify all metabolites in organisms, which could find the relationship between pathological and physiological changes and metabolites. It has been wildly used for clinical research, such as mechanism of diseases, biomarker discovery and prognosis prediction [7, 8]. Here, we used our previous reported targeted metabolomics profiling method to study the metabolites associated with vitamin D deficiency [9]. The method can quantify 289 metabolites of physiological significance in a single injection of 27 min without sacrificing sensitivity in both positive and negative scanning modes [9]. More than 97% of metabolites achieved similar sensitivity compared to the traditional separate positive and negative injections. Here we used this highly efficient detection method to establish metabolomics profiles relevant to vitamin D supplementation in pregnancy, helping to explore the mechanism of maternal vitamin D supplementation.

## Methods

### Chemicals

Isotopically labeled internal standards (ISs), such as valine-d8, thymine-d4, 17-hydroxyprogesterone-d8, cholic acid‑d4, docosahexaenoic acid‑d5, phenylalanine-d8, glycocholic acid‑d4 and chenodeoxycholic acid‑d4 were purchased from Cayman Chemical (Ann Arbor, MI, USA), Steraloids (Newport, RI, USA) or Chemical Cambridge Isotope Laboratories (Cambridge, MA, USA). High-performance liquid chromatography grade solvents including isopropanol, acetonitrile and formic acid were form Fisher Scientific (Pittsburgh, PA, USA). The rest of reagents were of analytical grade or higher. Unlabeled chemical standards were reported previously [9].

### Study design and participants

**Fig. 1:**
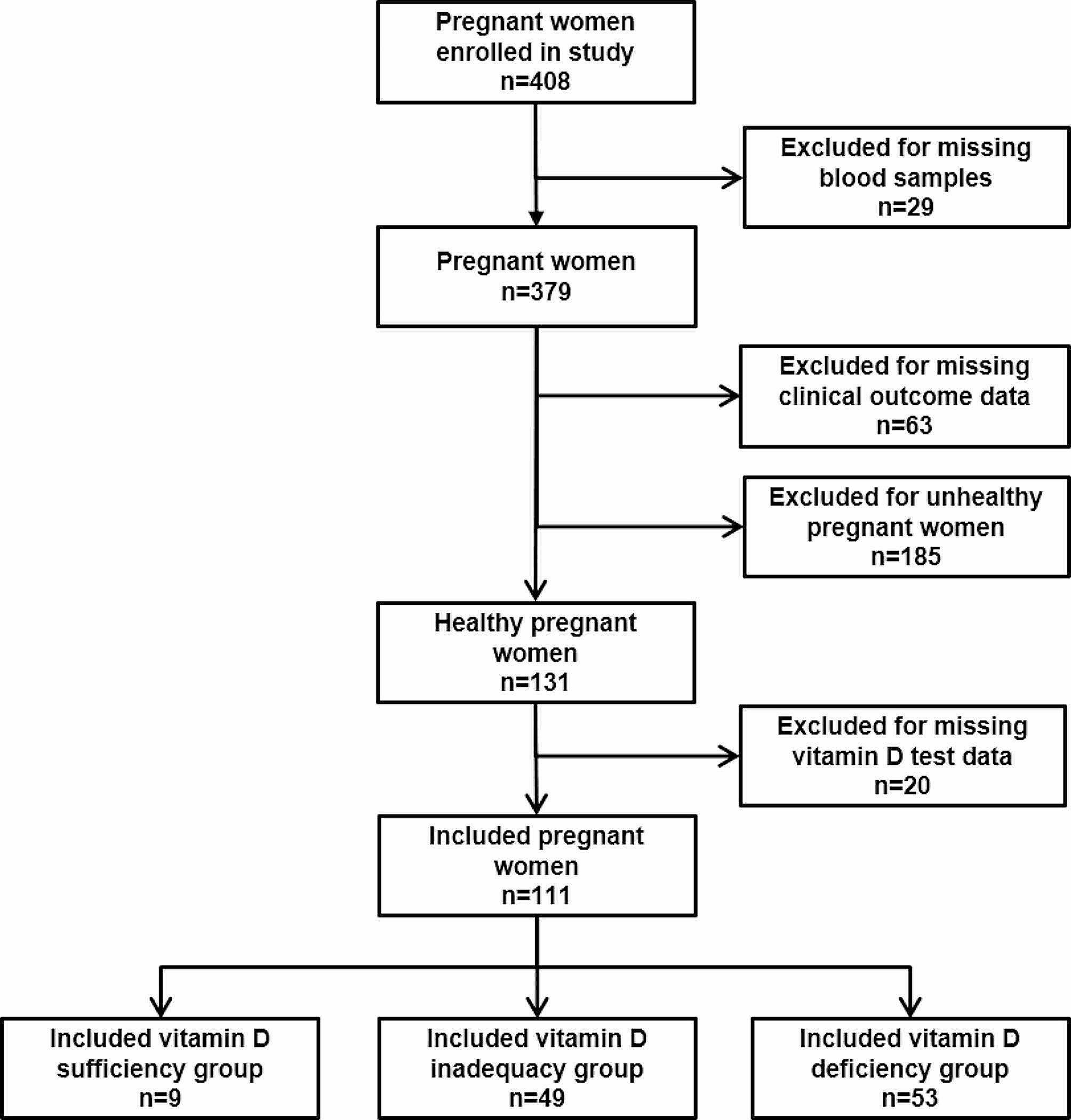
Participant flowchart for the metabonomics study of early pregnancy cohort in different vitamin D status (sufficient, inadequacy or deficiency)

In this study, 408 women were enrolled in their early pregnancy as 5 to 14 weeks of gestation from October to December 2018 in Shunyi District Maternal and Child Health Hospital (Beijing, China) (ClinicalTrials.gov registry, number NCT03651934). The remaining samples after physiological testing were used for research. The information including nutritional supplements and dietary intakes of all participants was collected. Twenty-nine participants were excluded for missing blood samples, 63 participants were excluded for missing clinical outcomes, 185 unhealthy participants were excluded (diagnosed with gestational diabetes mellitus, gestational hypertension, gestational heart disease, termination of pregnancy), and 20 participants were excluded for missing vitamin D test data. The remaining 111 pregnant women were divided into three group, including sufficient group (*n* = 9), inadequate group (*n* = 49) and deficient group (*n* = 53). The criteria was sufficient group ( vitamin D concentration >30 ng/ml), inadequate group (vitamin D concentration 20–30 ng/ml) and deficient group (vitamin D concentration <20 ng/ml) [10]. All the participants included in this study are natural conception. The nutritional supplement was standard intake of multivitamin tablet once every day (elevit®, BAYER). The study design is presented in Fig. 1.

### Sample preparation

In brief, the protein was precipitated by mixing 10 µL ISs mixture solution (400 ng/ml) with 50 µL serum and 140 µL MeOH (− 20 °C). The mixtures were prepared by centrifugation (13,800 g, 10 min, 4 °C) after vortex for 2 min o to get the supernatant. The supernatant was a collected for analysis.

### Mass spectrometry data acquiring and processing

Targeted metabolomics data was acquired using a Spark Holland liquid chromatography system (Spark, Holland) coupled with an API 5500 mass spectrometer (AB Sciex, Canada). This targeted metabolomics method covered a wide range of chemical categories, including 60 acyl carnitines, 42 nucleotides and derivatives, 11 bioamines and derivatives, 16 organic acids and derivatives, 20 sugar and derivatives, 33 bile acids, 54 amino acids and derivatives, 27 fatty acids, 13 steroids, 11 vitamins and derivatives, and 2 pteridines and derivatives. A HSS T3 (150 × 2.1 mm, 3.5 μm) was used for chromatographic separation. The autosampler temperature of column was set as 20 °C, the flow rate at 0.5 mL/min, and the injection volume at 5 µL. Mobile phase A contained water with 0.1% formic acid. Mobile phase B was acetonitrile: isopropyl (7:2, v: v). A gradient elution procedure was used, mobile phase A: 0–4 min, 99–90%; 4–8 min, 90–50%; 8–15 min, 50–20%; 15–25 min, 20–0%; 25–27 min, 0–0%.

The electrospray ionization source conditions were source temperature set at 600 °C, ion source gas1 set 60 psi, ion source gas2 at 60 psi. curtain gas at 30 psi, ion spray voltage floating (ISVF) at -4500 V and 5500 V for negative and positive mode respectively. The parameters including declustering potentials (DPs), collision energy (CE) and other were optimized automatically.

### Quality control

Four different concentrations (5, 50, 500 and 2000 ng/mL) of quality control (QC) samples were prepared from standard mixtures (including valine-d8, thymine-d4, 17-hydroxyprogesterone-d8, phenylalanine-d8, cholicacid‑d4, docosahexaenoic acid‑d5, glycocholic acid‑d4 and chenodeoxycholic acid‑d4 dissolved in methanol) with six duplicates. The average percentage difference between the measured and actual concentration of QC samples was set as accuracy. Relative standard deviation (RSD) of repeated measurements of six QC samples was set as precision.

The real quality assessment sample was prepared by mixing equal amount (10 µL) of the metabolites from the serum samples, and was used to assess the system’s stability and repeatability during real biological sample analysis.

### Statistical analysis

Peak extraction was conducted using MultiQuant software from AB SCIEX (version 3.0.2). The SIMCA 14.1 (Umetrics, Sweden) software was used to conduct principal component analysis (PCA). Student’s t-test was conducted using SPSS 21 (Armonk, New York, United States) to measure the significance of metabolites, and the *p* < 0.05 were considered significant. Jack knife-based confidence interval analyses were subsequently employed to remove variables with low reliability, and the differential metabolites were screened (variable importance fot the project, VIP > 1.0 and *p* < 0.05). Cluster and pathway analysis were performed using MetaboAnalyst 5.0 (Xia Lab @ McGill Sweden).

## Results

### Baseline data of patients

In this research, the baseline data of patients was showed in Table 1. There were no significant different parameters between the sufficient, inadequacy and deficiency groups according to the data collected, including maternal age, body mass index, gestation age, and hemoglobin or other parameters.

**Table 1:** Characteristics of the study cohorts

Parameter	vitamin D sufficient (*n* = 9)	vitamin D inadequacy (*n* = 49)	vitamin D deficiency (*n* = 53)
Maternal age, years	28.3 ± 2.57	28.82 ± 3.24	28.44 ± 2.80
Gestation age, days	67.1 ± 16.7	65.1 ± 16.8	68.167.1 ± 16.5
Education, n (%)			
Junior middle school	0 (0.0)	4 (8.2)	4 (7.5)
Technical/senior high school	9 (100.0)	19 (38.8)	23 (43.4)
Further education	0 (0.0)	22 (44.9)	23 (43.4)
Missing	0 (0.0)	4 (8.2)	3 (5.7)
History of polycystic ovarian syndrome, n (%)			
Yes	1 (11.1)	2 (4.1)	2 (3.8)
No	7 (77.8)	44 (89.8)	48 (90.6)
Missing	1 (11.1)	3 (6.1)	3 (5.7)
History of dyslipidemia, n (%)			
Yes	1 (11.1)	3 (6.1)	2 (6.7)
No	6 (66.7)	44 (89.8)	49 (92.5)
Missing	2 (22.2)	2 (4.1)	2 (6.7)
BMI at enrollment, kg/m^2^	21.20 ± 2.29	22.48 ± 3.47	22.59 ± 3.22
Hemoglobin, g/L	131.30 ± 7.04	130.10 ± 9.18	132.58 ± 8.84
Serum iron, µg/dL	117.64 ± 35.59	112.03 ± 39.01	123.14 ± 32.56
C-reactive protein, mg/L	2.79 ± 2.30	3.17 ± 3.17	2.48 ± 2.63
Glucose	4.38 ± 0.29	4.25 ± 0.71	4.44 ± 0.38
Alanine transaminase, U/L	20.5 ± 7.71	19.55 ± 40.60	47.24 ± 11.34
Total protein, g/L	70.81 ± 3.59	70.82 ± 3.71	70.72 ± 2.96
Total bilirubin, umol/L	11.92 ± 3.96	11.96 ± 4.78	13.22 ± 5.05
Alkaline phosphatase, u/l	47.60 ± 8.40	47.38 ± 13.16	47.24 ± 11.37
Creatinine, umol/L	49.00 ± 6.94	50.79 ± 27.30	46.36 ± 9.39
Homocysteine, umol/L	8.45 ± 1.34	8.46 ± 1.01	8.93 ± 1.06
serum folate ng/mL	22.82 ± 2.09	21.33 ± 3.74	18.52 ± 5.54
FT4(ng/dL)	0.91 ± 0.21	0.84 ± 0.14	0.81 ± 0.10
FT3(pg/mL)	3.04 ± 0.47	3.04 ± 0.58	2.87 ± 0.52
Vitamin D (ng/ml)	40.44 ± 20.42	24.62 ± 3.02	14.75 ± 3.81
Mg (mmol/L)	0.79 ± 0.06	0.84 ± 0.17	0.84 ± 0.15

### Lipid metabolic profiles in early pregnancy

**Fig. 2:**
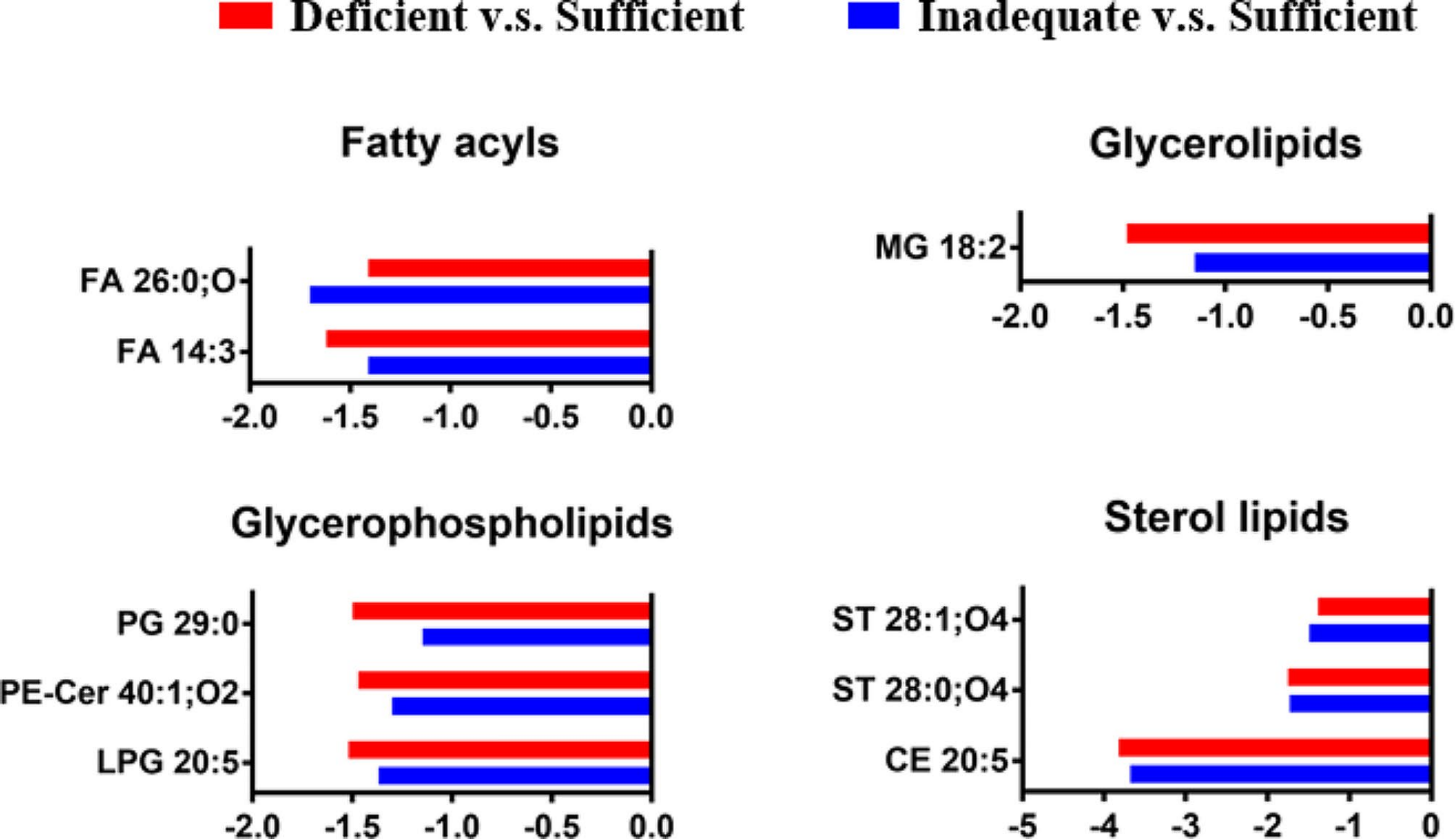
Fold change of different nonpolar metabolites found in serum from vitamin D deficient versus sufficient and inadequate versus sufficient group. The x-axis = fold change (FC): FC < 0 represents down-regulated metabolites in deficient or inadequate group. Abbreviations: fatty acyls, FA; sterol lipids, ST; Monoglycerol, MG; phosphatidylethanolamine, PE; cholesteryl ester, CE; phosphatidylglycerol, PG; long-chain phosphatidylglycerol, LPG; ceramide, Cer

The significance criteria was set as *p* < 0.05 and VIP > 1.0. There are 9 non-polar metabolites from four lipid categories (glycerolipids, GL; sterol lipids, ST; glycerophospholipids, GP; fatty acyls, FA) summarized in Fig. 2. The significant different lipid metabolites between vitamin D deficient versus sufficient and inadequate versus sufficient group are shown in Table 2. Two fatty acyls (FA), one glycerolipid (GL), three glycerophospholipids (GP) and three sterol lipids (ST) were down-regulated.

**Table 2:** Detailed information of significant differences in lipid metabolites between vitamin D deficient versus sufficient and inadequate versus sufficient group

Category	mz	Name	Formula	Deficiency versus Sufficiency			Inadequacy versus Sufficiency		
				Fold change	*P* value	Fold change		*P* value	
Fatty Acyls	221.1545	FA 14:3	C14H22O2	-1.62	1.70E-02	-1.41		1.63E-01	
	411.3853	FA 26:0;O	C26H52O3	-1.41	3.37E-02	-1.70		8.02E-03	
Glycerolipids	355.2837	MG 18:2	C21H38O4	-1.48	4.97E-02	-1.15		4.98E-01	
Glycerophospholipids	531.2735	LPG 20:5	C26H43O9P	-1.52	2.18E-02	-1.37		1.00E-01	
	743.6073	PE-Cer 40:1;O2	C42H85N2O6P	-1.47	2.68E-02	-1.30		1.91E-01	
	698.4978	PG 29:0	C35H69O10P	-1.50	3.73E-02	-1.15		5.84E-01	
Sterol lipids	688.6042	CE 20:5	C47H74O2	-3.82	4.03E-02	-3.68		3.43E-02	
	449.3648	ST 28:0;O4	C28H50O4	-1.75	4.52E-03	-1.73		7.56E-03	
	447.3487	ST 28:1;O4	C28H48O4	-1.38	1.86E-03	-1.49		3.79E-04	

### Polar metabolic profiling in early pregnancy

**Fig. 3:**
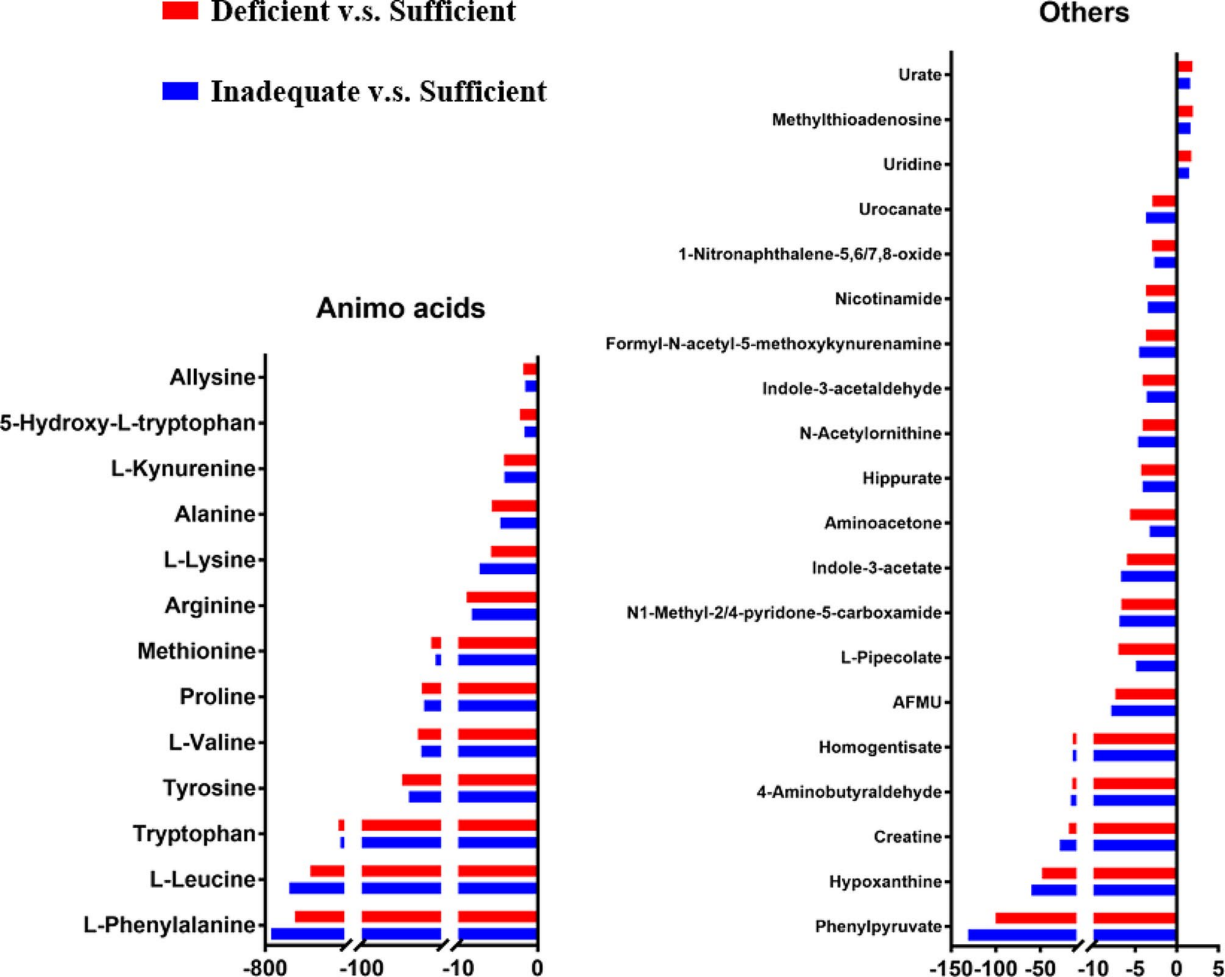
Fold change of different polar metabolites found in serum from vitamin D deficient versus sufficient and inadequate versus sufficient group. The x-axis = fold change (FC): FC > 0 represents up-regulated metabolites in deficient or inadequate group; FC < 0 represents down-regulated metabolites in deficient or inadequate group. Abbreviations: 5-Acetylamino-6-formylamino-3-methyluracil, AFMU.

There were 33 significant different polar metabolites (*p* < 0.05) in deficient and inadequate group, compared to the vitamin D sufficient cohort (Fig. 3). Many amino acids decreased, including phenylalanine, leucine, tryptophan, tyrosine, valine, proline, methionine, arginine, lysine, alanine, L-kynurenine,5-hydroxy-L-tryptophan, allysine. Besides, phenylpyruvate, hypoxanthine, creatine, 4-aminobutyraldehyde, homogentisate, AFMU, L-pipecolate, N1-methyl-2/4-pyridone-5-carboxamide, indole-3-acetate, aminoacetone, hippurate, N-acetylornithine, indole-3-acetaldehyde, formyl-N-acetyl-5-methoxykynurenamine, nicotinamide, 1-nitronaphthalene-5,6/7,8-oxide, urocanate also decreased. Only uridine, methylthioadenosine and urate increased. The changed metabolites are summarized in Table 3.

**Table 3:** Detailed information of significant differences in polar metabolites between vitamin D deficiency and sufficiency pregnant women

mz	Name	Formula	Deficiency versus Sufficiency			Inadequacy versus Sufficiency		
			Fold change	*P* value	Fold change		*P* value	
166.0858	Phenylalanine	C9H11NO2	-536.31	1.36E-02	-744.05		1.70E-02	
132.1016	Leucine	C6H13NO2	-398.83	1.39E-02	-584.41		1.74E-02	
205.0966	Tryptophan	C11H12N2O2	-152.61	1.37E-02	-134.47		1.73E-02	
182.0807	Tyrosine	C9H11NO3	-54.05	1.35E-02	-46.28		1.73E-02	
118.0859	Valine	C5H11NO2	-36.39	1.07E-02	-32.15		1.40E-02	
116.0705	Proline	C5H9NO2	-31.92	1.22E-02	-29.07		1.57E-02	
150.058	Methionine	C5H11NO2S	-21.03	1.12E-02	-16.03		1.57E-02	
175.1184	Arginine	C6H14N4O2	-8.91	7.42E-03	-8.29		1.05E-02	
147.1124	Lysine	C6H14N2O2	-5.88	1.40E-02	-7.31		1.34E-02	
90.0552	Alanine	C3H7NO2	-5.79	3.95E-03	-4.69		7.99E-03	
209.0913	L-Kynurenine	C10H12N2O3	-4.21	1.44E-02	-4.17		1.81E-02	
221.0914	5-Hydroxy-L-tryptophan	C11H12N2O3	-2.23	1.36E-02	-1.65		1.54E-01	
146.0809	Allysine	C6H11NO3	-1.80	5.31E-03	-1.57		2.30E-02	
165.0542	Phenylpyruvate	C9H8O3	-100.26	1.26E-02	-131.10		1.56E-02	
137.0455	Hypoxanthine	C5H4N4O	-48.14	1.35E-02	-59.90		1.64E-02	
132.0762	Creatine	C4H9N3O2	-18.40	6.93E-03	-28.54		7.54E-03	
88.076	4-Aminobutyraldehyde	C5H9NO2	-14.53	1.35E-03	-16.05		1.77E-03	
169.0491	Homogentisate	C8H8O4	-13.99	1.32E-02	-14.21		1.65E-02	
227.0783	AFMU	C8H10N4O4	-7.34	8.72E-03	-7.83		1.06E-02	
130.086	L-Pipecolate	C6H11NO2	-6.98	7.17E-03	-4.85		1.68E-02	
153.0655	N1-Methyl-2/4-pyridone-5-carboxamide	C7H8N2O2	-6.64	5.39E-03	-6.85		6.97E-03	
176.0701	Indole-3-acetate	C10H9NO2	-5.93	1.67E-03	-6.66		1.52E-03	
74.0606	Aminoacetone	C3H7NO	-5.60	1.04E-03	-3.22		3.09E-02	
180.065	Hippurate	C9H9NO3	-4.27	2.42E-02	-4.10		3.51E-02	
175.1074	N-Acetylornithine	C7H14N2O3	-4.11	1.83E-02	-4.63		1.64E-02	
160.0753	Indole-3-acetaldehyde	C10H9NO	-4.11	1.59E-02	-3.58		2.61E-02	
265.1169	Formyl-N-acetyl-5-methoxykynurenamine	C13H16N2O4	-3.70	4.16E-03	-4.50		2.69E-03	
123.0551	Nicotinamide	C6H6N2O	-3.68	1.45E-02	-3.45		2.16E-02	
190.0492	1-Nitronaphthalene-5,6/7,8-oxide	C10H7NO3	-2.97	3.44E-03	-2.68		1.44E-02	
139.0498	Urocanate	C6H6N2O2	-2.93	5.09E-03	-3.68		5.23E-04	
243.0618	Uridine	C9H12N2O6	1.72	3.09E-02	1.49		1.67E-01	
298.097	Methylthioadenosine	C11H15N5O3S	1.92	4.73E-02	1.66		8.33E-02	
167.0199	Urate	C5H4N4O3	1.86	4.17E-02	1.62		8.24E-02	

**Fig. 4:**
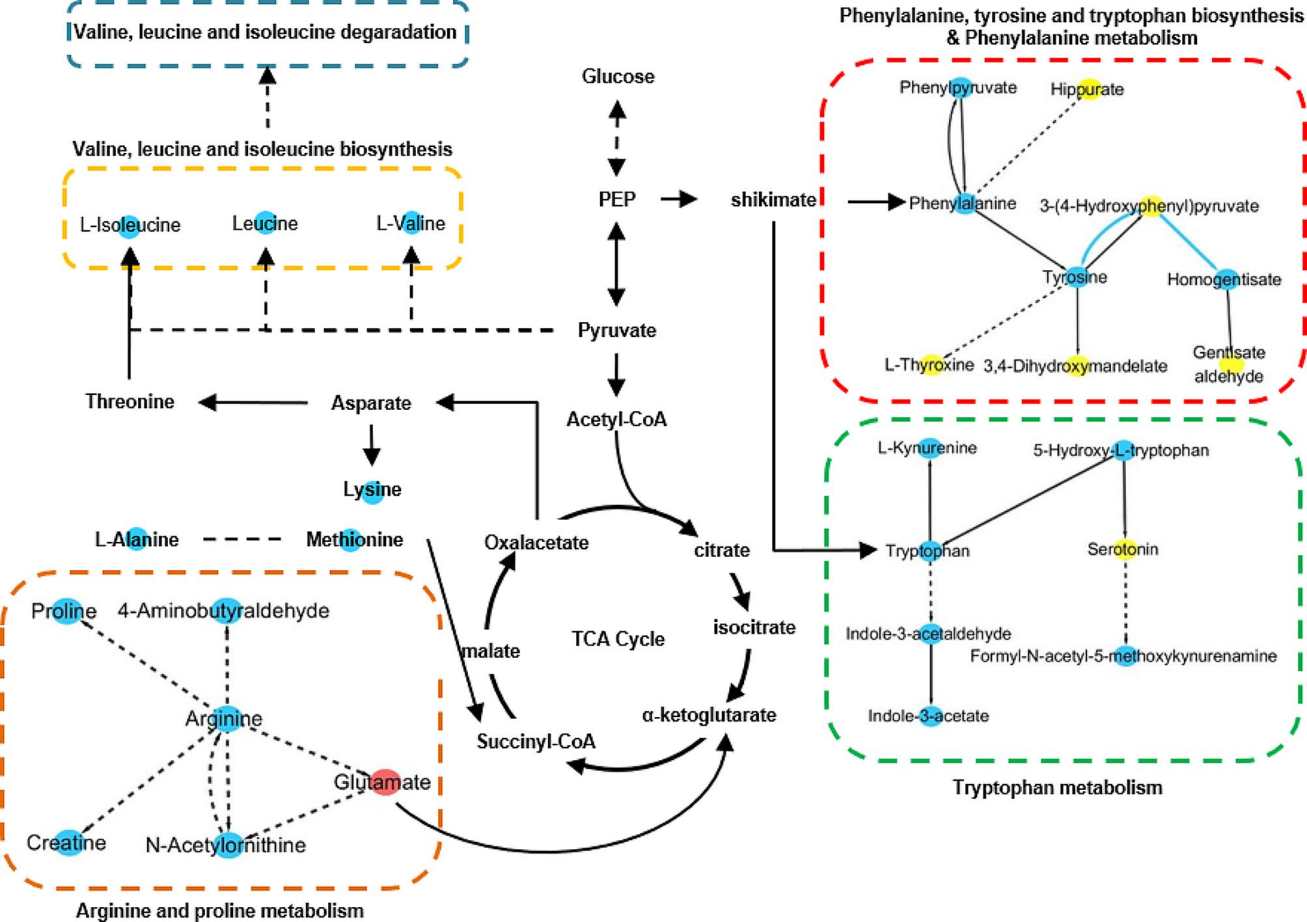
Metabolic enriched activity network of serum polar metabolites in vitamin D deficient pregnant women. Red/blue nodes represent higher/lower intensity in vitamin D deficient pregnant women. Yellow nodes represent similar levels of metabolites between vitamin D deficient versus sufficient samples. Blue edges represent the enzyme(s) between two metabolites that were inactivated. Metabolites that had no direct relationship were linked by dotted lines. Black dashed edges represent activity of enzyme(s) without statistical significance

Ratio changes to the network reaction were analyzed, and a significant adjustment for two reactions were related to the activity of metabolic enzyme(s) between substrates and products (*p* < 0.05). The software Cytoscape was used to visualize significant different metabolic networks. The tyrosine/4-hydroxyphenylpyruvate ratio was down-regulated 26.18-fold (*p* = 0.010) by phenylalanine dehydrogenase, pyruvate aminotransferase and 2-oxoglutarate aminotransferase. The homogentisate/4-hydroxyphenylpyruvate ratio was down-regulated 7.58-fold (*p* = 0.007) by 4-hydroxyphenylpyruvate dioxygenase. The observed state of identified metabolites and metabolic enzymes are shown in the metabolic networks presented in Fig. 4.

There were five pathways enriched in the network, including phenylalanine, tyrosine and tryptophan biosynthesis, phenylalanine metabolism, tryptophan metabolism, arginine and proline metabolism, valine, leucine and isoleucine biosynthesis. All these metabolic pathways were related to amino acid metabolism in the activity network.

## Discussion

Here, we used LC-HRMS method to acquire metabolic profiles associated with VD level.

during pregnancy. This cohort enrolled 111 pregnant women, including sufficient group (*n* = 9), inadequate group (*n* = 49) and deficient group (*n* = 53). We divided the test population into three groups based on the concentration of vitamin D. The criteria was sufficient group ( vitamin D concentration >30 ng/ml), inadequate group (vitamin D concentration 20–30 ng/ml) and deficient group (vitamin D concentration <20 ng/ml) [10]. In this manuscript, we are trying to draw a differential metabolite map, aiming to help elucidating the relation between vitamin D and pregnant women health. Therefore, we compared the 9 subjects of the sufficient group with deficient and inadequate groups. As show in Figs. 2, 3 and 4, the changed degree difference of deficient group is obviously greater than inadequate group. Two fatty acyls (FA), one glycerolipid (GL), three glycerophospholipids (GP) and three sterol lipids (ST) were down-regulated.

Glycerophospholipids have important effects on the health of pregnant and postpartum women. Glycerophospholipids are essential components of biological membranes, playing a crucial role in maintaining the structure and function of these membranes. During pregnancy, glycerophospholipids serve as an energy source, providing necessary nutrients to the fetus. Additionally, they protect both the fetus and the mother from oxidative stress and inflammatory responses. Glycerophospholipids can inhibit cell apoptosis and necrosis, promoting cell proliferation and differentiation. This helps maintain normal placental function and fetal development. Although the specific relationship between vitamin D and glycerophospholipids is currently unclear, maintaining appropriate levels of vitamin D is crucial for the health of pregnant women and their fetuses [11].

Sterol lipids are one of the important nutrients required for fetal development. They also participate in immune regulation and anti-inflammatory responses in pregnant women. Abnormal levels of sterol lipids in pregnant women may have adverse effects on maternal and fetal health. For example, hypercholesterolemia can increase the risk of complications such as gestational diabetes and hypertension in pregnant women. Additionally, high sterol levels may also adversely affect fetal development. Gholamzad A et al. found that as the serum level of vitamin D increased, the mean low-density lipoprotein (LDL) level decreased significantly in a cross-sectional study of 15,600 patients, suggesting that vitamin D status may play a role in regulating lipid metabolism. The mechanism may be that vitamin D could increase the production of bile salts and reducing the activity of lecithin-cholesterol acyltransferase [12]. Singh P et al. found that vitamin D supplementation could increase gut microbiome diversity. After vitamin D supplementation, genes abundance involved in metabolism of amino acids and lipids elevated, especially fatty acid elongation and steroid biosynthesis. This may be that lipids, bile salts and fatty acids are main components of mixed micelles, which act as a prerequisite for fat-soluble vitamin D absorption. So, the increased bacterial gene abundance related to fatty acid and lipid metabolism after vitamin D supplementation could indicate positive impact of vitamin D supplementation on pregnant women [13]. Therefore, pregnant women should maintain appropriate levels of sterol lipids during pregnancy and regularly undergo prenatal check-ups to monitor blood lipid levels and other indicators [14, 15].

Many amino acids decreased in vitamin D deficient and inadequate groups, including aromatic amino acids (tryptophan, phenylalanine, tyrosine) and branched-chain amino acids (valine, isoleucine, leucine), proline, methionine, arginine, lysine, alanine, L-kynurenine,5-hydroxy-L-tryptophan, allysine.

Mieszkowski J et al. found that vitamin D supplementation can significantly affect exercise-induced changes in tryptophan metabolism and kynurenine metabolism, suggesting vitamin D supplementation could suppress the protein degradation during sustained exercise [16–18]. Some studies also have shown that vitamin D deficiency may lead to muscle atrophy and muscle strength decline, which may be related to abnormalities in amino acid metabolism. The underlying mechanisms may be multifaceted. Specifically, vitamin D deficiency may affect the uptake and utilization of branched-chain amino acids (BCAA) by muscles, thereby affecting muscle synthesis and catabolism. Additionally, vitamin D may also affect the activity of other hormones and enzymes related to amino acid metabolism, further affecting amino acid metabolism. To maintain healthy amino acid metabolism, pregnant women need to maintain appropriate levels of vitamin D. Foods rich in vitamin D include fish, egg yolks, dairy products, and so on [19, 20].

The fetus obtains vitamin D from the mother through the placenta, and the amount of vitamin D in the newborn is related to the maternal vitamin D nutritional status and gestational age. Insufficient vitamin D storage can occur when the mother is undernourished during pregnancy, especially in the latter part of pregnancy, such as malnutrition, liver and kidney disease, chronic diarrhea, premature birth, and twin births. The majority of pregnant women have vitamin D deficiency or deficiency, which results in inadequate supply of vitamin D to meet the needs of their newborns in the first 2 weeks of life. Therefore, most countries recommend starting vitamin D supplementation in the first few days after birth.

This project also has some limitations: (1) the sample size is relatively small since we did not have a large cohort in our single center. (2) the mechanism needs to be further studied in a mouse modle.3) lacking successive stages beyond early pregnancy. In summary, there is still a long way before the findings become more reproducible for proof and clinical application.

## Data Availability

Additional data can on request be made available. Contact information: Xiaogang Li, email: lxgang8810@163.com.
